# Rare-Earth-Metal (Nd^3+^, Ce^3+^ and Gd^3+^)-Doped CaF_2_: Nanoparticles for Multimodal Imaging in Biomedical Applications

**DOI:** 10.3390/pharmaceutics14122796

**Published:** 2022-12-14

**Authors:** Zhenfeng Yu, Yuanyuan He, Timo Schomann, Kefan Wu, Yang Hao, Ernst Suidgeest, Hong Zhang, Christina Eich, Luis J. Cruz

**Affiliations:** 1 Translational Nanobiomaterials and Imaging Group, Department of Radiology, Leiden University Medical Center, 2333 ZA Leiden, The Netherlands; 2 Percuros B.V., Zernikedreef 8, 2333 CL Leiden, The Netherlands; 3 Van ‘t Hoff Institute for Molecular Sciences, University of Amsterdam, Science Park 904, 1098 XH Amsterdam, The Netherlands; 4 C.J. Gorter Center for High Field MRI, Department of Radiology, Leiden University Medical Center, 2333 ZA Leiden, The Netherlands

**Keywords:** multimodal imaging, NIR-II, PAI, MRI, rare-earth-doped nanoparticles

## Abstract

Here, we describe the synthesis of a novel type of rare-earth-doped nanoparticles (NPs) for multimodal imaging, by combining the rare-earth elements Ce, Gd and Nd in a crystalline host lattice consisting of CaF_2_ (CaF_2_: Ce, Gd, Nd). CaF_2_: Ce, Gd, Nd NPs are small (15–20 nm), of uniform shape and size distribution, and show good biocompatibility and low immunogenicity in vitro. In addition, CaF_2_: Ce, Gd, Nd NPs possess excellent optical properties. CaF_2_: Ce, Gd, Nd NPs produce downconversion emissions in the second near-infrared window (NIR-II, 1000–1700 nm) under 808 nm excitation, with a strong emission peak at 1056 nm. Excitation in the first near- infrared window (NIR-I, 700–900 nm) has the advantage of deeper tissue penetration power and reduced autofluorescence, compared to visible light. Thus, CaF_2_: Ce, Gd, Nd NPs are ideally suited for in vivo fluorescence imaging. In addition, the presence of Gd^3+^ makes the NPs intrinsically monitorable by magnetic resonance imaging (MRI). Moreover, next to fluorescence and MR imaging, our results show that CaF_2_: Ce, Gd, Nd NPs can be used as imaging probes for photoacoustic imaging (PAI) in vitro. Therefore, due to their biocompatibility and suitability as multimodal imaging probes, CaF_2_: Ce, Gd, Nd NPs exhibit great potential as a traceable imaging agent in biomedical applications.

## 1. Introduction

Imaging holds a crucial role in the diagnosis of a variety of diseases such as cancer. Early-stage disease diagnosis is important to maximize treatment effects, and to personalize treatments based on the patient’s individual variability and medical profile. Molecular imaging techniques provide comprehensive anatomical, physiological and functional information on disease detection and the monitoring of treatment responses. The most commonly used diagnostic imaging methods during the past few decades in the medical field include MRI, X-ray computed tomography (CT) [1], positron emission tomography (PET) [2,3], single-photon emission tomography (SPECT) [4], optical fluorescent light imaging (FLI) and photoacoustic imaging (PAI) [5]. Due to differences in their detection methods, spatiotemporal resolution, sensitivity and probe types, the diagnostic information obtained is divergent. Both PET and SPECT use γ rays to detect the in vivo distribution of radioactive tracers to obtain information on biological functions. They have the disadvantages of low spatial resolution, radiation risks and high costs [6,7,8,9,10]. Optical imaging uses visible light and near-infrared probes with different spectral characteristics for molecular and cellular detection but faces several limitations, such as photobleaching, low tissue penetration power, low spatial resolution and autofluorescence [11,12,13,14,15]. While CT, MRI and PAI can provide structural information, CT detection relies on contrast agents (such as iodine or barium) to obtain images through the different absorption of X-rays by biological tissues, which has the disadvantage of radiation risk and limited soft-tissue resolution [16,17]. MRI, a non-invasive imaging technology uses radio waves (magnetic field), but it has low sensitivity, high cost and scanning and image processing are time-consuming [18,19,20]. PAI uses high-frequency sound waves (>20 kHz) to generate acoustic energy to detect the difference in echo between chromophores or microbubbles and surrounding tissues in real time. However, due to the limited resolution and sensitivity, the data reproducibility is low, and it cannot provide accurate results [8,21,22,23]. In summary, to predict and treat diseases more comprehensively and accurately, it is imperative to develop a simple and efficient multifunctional nanomaterial that can integrate multiple imaging modes for detection.

Lanthanides display attractive photophysical properties, such as large anti-Stokes shift, narrow band and multiple emission and long-life luminescence due to their unique 4f^n^ structure and are widely used in the development of optical probes [24,25,26,27]. Nd^3+^-doped nanoprobes are downconversion NPs (DCNPs) ranging from NIR-I to NIR-II. They can emit at 890, 1060 and 1340 nm under 808 nm excitation and therefore can be used for near-infrared two-zone imaging [28,29]. Of note, due to the Stokes shift fluorescence mechanism of Nd^3+^, Nd^3+^-doped NPs have a larger quantum yield than upconversion NPs (UCNPs), and the absorption cross-section of Nd^3+^ at 808 nm is larger than the absorption cross-section of Yb^3+^ at 980 nm, which leads to little absorption by water molecules at 808 nm when using Nd^3+^-doped nanoprobes. Therefore, compared to Yb^3+^- doped UCNPs, the overheating effect of biological tissues can be more effectively avoided in bioimaging [30]. Because of the many advantages of Nd^3+^, Nd^3+^-doped NPs are undoubtedly an optical imaging probe with great potential that can be used for biosensing, biological process monitoring and imaging-guided therapy [31].

Another key to obtaining high-quality Nd^3+^-doped nanomaterials is to choose a suitable host matrix material. CaF_2_ is a center-symmetric fluorite crystal with low phonon energy and a wide band gap (E g = 12.1 eV). It is optically transparent in the wavelength range from ultraviolet to visible light and near infrared, so it is widely used in various types of optical equipment [32,33,34,35,36,37]. In addition, its unique fluorite structure can be easily doped with lanthanides. Under the condition of not destroying the main configuration, the presence of lanthanides causes internal valence changes and diversified charge compensation, resulting in a lattice structure with rich symmetry. These changes give the lanthanide-doped CaF_2_ crystals strong optical activity in the visible and near-infrared regions [38,39]. Thus, CaF_2_ can be used as an optical matrix material for Nd^3+^-doped nanomaterials.

Since the radius of Nd^3+^ is not much different from that of Ca^2+^, trivalent Nd ions replace the crystal sites of divalent Ca ions when they are doped into the CaF_2_ lattice, requiring more F^−^ for charge compensation, but they do not cause obvious crystal changes. However, when the Nd^3+^ doping concentration reaches a certain limit, Nd^3+^ aggregates and some energy cross-relaxation occurs, which triggers the cluster effect and reduces the luminous efficiency of the nanomaterials [40,41]. In order to overcome this phenomenon, the introduction of optically inactive ions (such as Lu^3+^, Gd^3+^, Y^3+^, Yb^3+^) can effectively destroy the formation of Nd-Nd clusters, thereby improving the quantum efficiency of the material [42,43,44,45]. Notably, the special properties of certain optically inactive ions also offer the possibility of constructing multimodal probes; however, most current multimodal imaging probes are dual-mode probes, and NIR-II-based imaging systems combining more than two modes are still rarely reported, but our previous study demonstrates that triple-mode imaging probes hold great promise for obtaining complementary information. Therefore, we focus on the field of rare-earth triple-mode imaging probes for more exploration [46]. Wang et al. showed that the luminous efficiency of CaF_2_: Nd co-doped Ce^3+^ was 10.4 times higher than that of CaF_2_: Nd [47]. On the other hand, the seven unpaired electrons of Gd(III) can shorten the proton relaxation rate, making Gd^3+^ a common T1 contrast agent in MR bioimaging [48,49]. Therefore, we choose Ce^3+^, Gd^3+^ and Nd^3+^ co-doping CaF_2_ as the material in our strategy to construct an imaging mode combining NIR-II imaging with complementary MRI and PAI.

In this study, we used a hydrothermal method to synthesize a citric acid-terminated nanomaterial with CaF_2_ as the matrix, co-doped with the three lanthanide elements—Ce, Gd and Nd. The resulting CaF_2_: Ce, Gd, Nd NPs had a small particle size, were stable in the presence of serum and showed excellent luminous intensity at 1056 nm. In vitro cell culture experiments demonstrated that CaF_2_: Ce, Gd, Nd NPs did not induce cellular cytotoxicity and were readily taken up by human breast cancer cells. In addition, the NPs showed low immunogenicity when cultured with antigen-presenting cells, and thus the NPs can be used as a potential imaging probe in vivo. Importantly, CaF_2_: Ce, Gd, Nd NPs are multimodal and can be detected by NIR-II/PA/MR imaging and therefore provide significant advantages in disease diagnosis. In summary, CaF_2_: Ce, Gd, Nd NPs represent a novel optical material that can be widely used in the field of medical imaging.

## 2. Materials and Methods

### 2.1. Materials

The following chemicals were acquired from Sigma-Aldrich (St. Louis, MO, USA): neodymium (III) chloride hexahydrate (NdCl_3_·6H_2_O, 99.9%), calcium chloride dihydrate (CaCl_2_·2H_2_O, 99.5%), cerium (III) chloride heptahydrate (CeCl_3_·7H_2_O, 99.9%), potassium citrate tribasic monohydrate (HOC(COOK)(CH_2_COOK)_2_·H_2_O, ≥99.0%), gadolinium (III) chloride hexahydrate (GdCl_3_·6H_2_O, 99.9%), ammonium fluoride (NH_4_F, ≥98.0%). Biolegend (San Diego, CA, USA) supplied the anti-CD40-APC. Thermo Fisher Scientific (Waltham, MA, USA) provided fetal calf serum (FCS), 4′,6-Diamidino-2-Phenylindole (DAPI), dulbecco’s Modified Eagle’s Medium (DMEM) and CD86-FITC. Promega (Madison, WI, USA) offered cell titer 96 AQueous MTS Reagent Powder. PeproTech (Cranbury, NJ, USA) supplied lipopolysaccharide (LPS). Bioline (London, UK) delivered agarose. Abcam (Cambridge, UK) supplied the phalloidin- iFluor 488 Reagent. All of water used in the experiments was ultrapure deionized water.

### 2.2. Synthesis of the CaF_2_: Ce, Gd, Nd NPs

As previously described [50], we synthesized CaF_2_: Ce, Gd, Nd NPs by a simple hydrothermal method. Briefly, a total of 3.75 mmol of CaCl_2_·2H_2_O, CeCl_3_·7H_2_O, GdCl_3_·6H_2_O and NdCl_3_·6H_2_O (Ca_0.98−2x_Ce_x_Gd_x_Nd_0.02_F_2.02+2x_, x = 0.15) were dissolved in 7 mL water and stirred for 10 minutes (min) until fully dissolved. Then, potassium citrate solution was added dropwise to the solution and stirred for 30 min. After that, NH_4_F solution was added, and the solution was stirred evenly. The final solution was transferred to a 50 mL Teflon bottle (Baoshishan, China) held in a stainless-steel autoclave, put in an oven (Heraeus, Germany) and maintained at 180 °C for 10 hours (h). Finally, the obtained sample was centrifuged at 2.4× *g* for 20 min and washed three times with water followed by ethanol (99%). The samples were dried in a freeze-dryer (Martin Christ, Osterode, Germany).

The synthesis of CaF_2_: Nd NPs, CaF_2_: Ce, Nd NPs, CaF_2_: Ce, Gd NPs was similar to that of CaF_2_: Ce, Gd, Nd NPs. The doped concentration of Nd^3+^ was maintained at 0.02, and Ce^3+^ and Gd^3+^ were maintained at 0.15.

### 2.3. Characterization

X-ray diffraction (XRD) analysis of the CaF_2_: Ce, Gd, Nd NPs was performed by a Panalytical X’pert PRO (Malvern Panalytical, Malvern, UK) operating at a tube voltage of 40 kV and a tube current of 40 mA. The diffraction patterns were acquired using Cu Kα radiation (λ = 1.5405 Å) at a scanning rate of 6.0°/min in the 2θ range of 10° ≤ 2θ ≤ 70°.

The hydrodynamic size and Zeta potential were obtained from CaF_2_: Ce, Gd, Nd NPs solution (1 mg/mL) using a Malvern ZetaSizer 2000 (Malvern, UK). The analysis software applications were Zetasizer Software (Version 7.13) and GraphPad Prism 8.

IRSpirit FTIR spectrophotometer (Shimadzu, Kyoto, Japan) was used to measure the Fourier transform infrared (FTIR) spectra of CaF_2_: Ce, Gd, Nd NPs powder. The spectra were captured in the IRSpirit-TOAPC0027956 mode with a wavenumber range of 600–4000 cm^−1^ and a resolution of 4 cm^−1^. IR Pilot and Origin 8.5 were used for analysis.

The size and morphology of CaF_2_: Ce, Gd, Nd NPs were examined using a Tecnai 12 Twin transmission electron microscope (TEM) (FEI Company, Hillsboro, OR, USA) outfitted with a OneView Camera Model 1095 (Gatan, Pleasanton, CA, USA) under 120 kV voltage. TEM samples were prepared by pipetting CaF_2_: Ce, Gd, Nd NPs aqueous solution (1 mg/mL) onto the surface of the copper grid.

CaF_2_: Ce, Gd, Nd NPs were mounted on scanning electron microscopy (SEM) specimen stubs. Then, an Apreo S LoVac SEM (Thermo Scientific, Waltham, MA, USA) equipped with an UltraDry energy-dispersive X-ray spectroscopy (EDS) detector (Thermo Scientific, Waltham, MA, USA) was utilized to analyze the sample. The measurement conditions were: 1500× magnification with 30 kV and 51 nA.

A Quantum Design Versalab physical property measurement system with VSM option (Quantum Design, San Diego, CA, USA) was employed to quantify the vibrating-sample magnetometry (VSM) of CaF_2_: Ce, Gd, Nd NPs (4.51 mg).

The emission spectra of CaF_2_:Ce, Gd, Nd NPs powder was recorded using Fluorolog^®^-3 with FluoEssence^TM^ (Horiba, Kyoto, Japan) equipped with a diode laser as the excitation light source, and the emission spectra of CaF_2_: Nd NPs (powder) and CaF_2_: Ce, Nd NPs (powder) were recorded using an Edinburgh FLS920 fluorescence spectrometer (Edinburgh Instruments, Edinburgh, UK) with an 808 nm NIR diode laser (300 mW). The absorption spectra of CaF_2_: Ce, Gd, Nd NPs (10 mg/mL) and CaF_2_: Ce, Gd NPs (10 mg/mL) were obtained by SpectraMax^®^ iD3 Multi-Mode Microplate Reader (Molecular Devices, San Jose, CA, USA). Analysis tool was SoftMax Pro^®^ 7 Software.

### 2.4. Stability of CaF_2_:Ce, Gd, Nd NPs

To assess the stability of CaF_2_: Ce, Gd, Nd NPs in physiologically relevant buffers, we dissolved CaF_2_: Ce, Gd, Nd NPs at a concentration of 0.2 mg/mL in 50% FCS solution. The samples were kept in a shaker at 37 °C, and we used a Malvern ZetaSizer 2000 (Malvern, UK) to measure the size and zeta potential at 0 h, 1 d, 2 d, 3 d, 4 d, 5 d, 6 d and 7 d. Data analysis used Zetasizer Software (Version 7.13) and GraphPad Prism 8.

### 2.5. Hemolysis of CaF_2_: Ce, Gd, Nd NPs

Briefly, a total of 100 μL of fresh blood was collected from the tail vein of BALB/c mice using a vacuum blood collection tube. Ca^2+^/Mg^2+^-free PBS was used to dilute the blood 50 times. To extract red blood cells, the blood dilutions were centrifuged at 0.9× *g* for 15 min at 4 °C. The supernatant was removed, and two groups were chosen randomly as negative and positive controls. The negative control group was resuspended with 500 μL of saline, whereas the positive control group was resuspended with 500 μL of 1% Triton X-100 (*v*/*v*). For the experimental group, blood cells were resuspended with CaF_2_: Ce, Gd, Nd NPs isotonic saline at five different concentrations (1, 0.5, 0.25, 0.125, 0.0625 mg/mL). The samples were then incubated at 37 °C for 4 h before being centrifuged at 0.9× *g* and 4 °C for 15 min to obtain the supernatant. The absorbance values were measured at 540 nm by an enzyme marker SpectraMax (Molecular Devices, San Jose, CA, USA). The concentration of cytosolic hemoglobin in each sample was assessed according to the hemoglobin concentration standard curve.

The hemolysis ratio was calculated using the hemolysis (HL%) equation follows:

H (hemolysis ratio, %) = (OD sample − OD negative)/(OD positive − OD negative) × 100

### 2.6. MTS Cytotoxicity Assay of CaF_2_: Ce, Gd, Nd NPs

The toxicity of CaF_2_: Ce, Gd, Nd NPs to cells was assessed by MTS assay using two different cell lines. Briefly, 4T1 cells (1 × 10^4^) were seeded in 96 wells and incubated for 24 h at 37 °C. The cells were then treated for 24, 48 and 72 h with varied concentrations of CaF_2_: Ce, Gd, Nd NPs (0–2000 µg/mL). Meanwhile, a 96-well plate was inoculated with 1 × 10^5^ peripheral blood mononuclear cells (PBMCs)/well, kept in a 37 °C incubator and then incubated with various CaF_2_: Ce, Gd, Nd NPs concentrations (0–125 µg/mL) for 24 h, 48 h and 72 h. Afterwards, the medium was removed and 100 µL of fresh medium and 20 μL of MTS reagent were added to each well as directed by the manufacturer’s instructions. The cells were then cultured for 1.5 h at 37 °C in an incubator. The absorbance (OD) was measured using Microplate reader (Molecular Devices, San Jose, CA, USA) at 490 nm. The ratio to the untreated control group was used to assess cell viability. Data are presented as mean ± SD.

### 2.7. Uptake of CaF_2_: Ce, Gd, Nd NPs

Qualitative uptake of CaF_2_: Ce, Gd, Nd NPs in 4T1 cells was determined by confocal microscopy. 4T1 cells were seeded at 2 × 10^4^/well on 13 mm^3^ circular coverslips in 24-well plates. After 24 h, 4T1 cells were co-incubated with 250 µg/mL of CaF_2_: Ce, Gd, Nd NPs medium at 37 °C for 1, 4, 24 and 48 h. Cells treated with medium without NPs served as a control group. At the end of the incubation time, the cells were washed 5 times with PBS to remove the unabsorbed NPs. At room temperature, the cells were fixed with 4% paraformaldehyde for 15 min, washed twice with PBS, treated with 0.1% Triton PBS for 10 min and washed 3 times with PBS. Finally, cell nuclei were labeled with DAPI for 5 min. After washing with PBS, coverslips were sealed on slides with fluorCare sealer. The slides were imaged using a SP8 LIGHTNING Confocal Microscope (Leica Biosystems Nussloch GmbH, Germany) and analyzed by LAS X (Leica Application Suite X) software.

### 2.8. In Vitro Dendritic Cell (DC) Activation Study

In order to evaluate the effect of CaF_2_: Ce, Gd, Nd NPs on immune cells, we used flow cytometry to assess the expression of DC maturation/activation markers. Briefly, murine D1 DCs and 125 µg/mL CaF_2_: Ce, Gd, Nd NPs were co-cultured in a 96-well plate for 24 h in a 37 °C incubator. PBS/EDTA (Sigma-Aldrich, St. Louis, MO, USA) was used to detach the cells, which were then washed with FACS buffer and stained with anti-CD40-APC (Biolegend, San Diego, CA, USA) and anti-CD86-FITC (eBioscience, San Diego, CA, USA) antibodies. The cells were washed and resuspended in 100 μL FACS buffer after 30 min. A LSR-II cytometer (BD Biosciences, Franklin Lakes, NJ, USA) was used to measure the samples, and FlowJo (version 10) was utilized to analyze the data.

### 2.9. Multimodal Imaging Properties of CaF_2_: Ce, Gd, Nd NPs

#### 2.9.1. NIR-II imaging

We employed an in vivo NIR-II optical imaging system (Kaer Labs, Nantes, France) to evaluate the NIR-II imaging capabilities of our NPs. CaF_2_: Ce, Gd, Nd NPs were suspended in ddH_2_O at 2 mg/mL, while the InGaAs camera was chilled to −20 °C at mid-gain setup. We obtained photos at various wavelengths using an 808 nm laser excitation at 50 mW/cm^2^. Images were recorded with the KIS NIR-II system.

#### 2.9.2. Photoacoustic Imaging

The PA and B-mode ultrasound images were acquired using Vevo LAZR-X (FUJIFILM VisualSonics, Toronto, ON, Canada), and a MX550D transducer was utilized. CaF_2_: Ce, Gd, Nd NP solution was injected into an 0.5% agarose gel. The experiment was carried out with the center transmit of 40 MHz and the axial resolution of 40 μm. Vevo LAB 5.5.0 was used to analyze the data.

#### 2.9.3. MRI Studies

MRI studies were carried out using a 7T Bruker BioSpec (Ettlingen, Germany), and ParaVision 360 (Version 2.0. pl.1) software was used to analyze attenuation images. CaF_2_: Ce, Gd, Nd NPs were dissolved in 0.5% agarose solution, and the concentrations were 0 mg/mL, 1 mg/mL, 2.5 mg/mL, 5 mg/mL, 7.5 mg/mL and 10 mg/mL. The microwave oven was used to heat the solutions and create the gel sample. The measurement settings were as follows: 500/11 ms for the repetition time/echo time (TR/TE), 4 for the number of excitations (NEX), 128 × 128 for the matrix size (MTX), 40 × 40 mm^2^ for the field of view (FoV) and 1.20 mm for the slice thickness (SL). To investigate the MRI characteristics of NPs under biological conditions, we fixed C57BL/6J mouse cadavers with a tiny animal coil after subcutaneously injecting 100 μL of CaF_2_: Ce, Gd, Nd NPs (10 mg/mL) and took MR images before and after the injection. The measurement conditions were TR/TE = 10/2.8 ms, FoV = 40 × 40 mm^2^, matrix = 256 × 256.

### 2.10. Statistical Analysis

The statistical tests performed by GraphPad Prism software 8 (GraphPad Software, San Diego, CA, USA). Statistical differences were considered significant at * *p* < 0.05, ** *p* < 0.01, *** *p* < 0.001 and **** *p* < 0.0001.

## 3. Results and Discussion

**Scheme 1:**
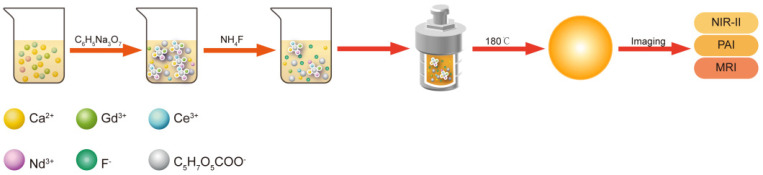
Synthesis process and applications of CaF_2_: Ce, Gd, Nd NPs.

To explore whether our strategy of synthesizing a multimodal citric acid-terminated nanomaterial with CaF_2_ as the matrix, co-doped with the three lanthanide elements—Ce, Gd and Nd—was feasible, we adapted a previously published hydrothermal method to obtain Yb^3+^/Tm^3+^-doped MF_2_ (M = Ca, Sr) colloids to synthesize CaF_2_: Ce, Gd, Nd. Briefly, Ca^2+^, Ce^3+^, Gd^3+^, Nd^3+^ were dissolved in water. Next, solutions of potassium citrate tribasic monohydrate and NH_4_F were added in turn to obtain the final solution. The resulting solution was heated in an oven (Heraeus, Germany) at 180 °C for 10 h to synthesize NPs. The obtained sample was centrifuged, washed and freeze-dried (Scheme 1). The physicochemical properties of the synthesized NPs were carefully characterized.

**Figure 1:**
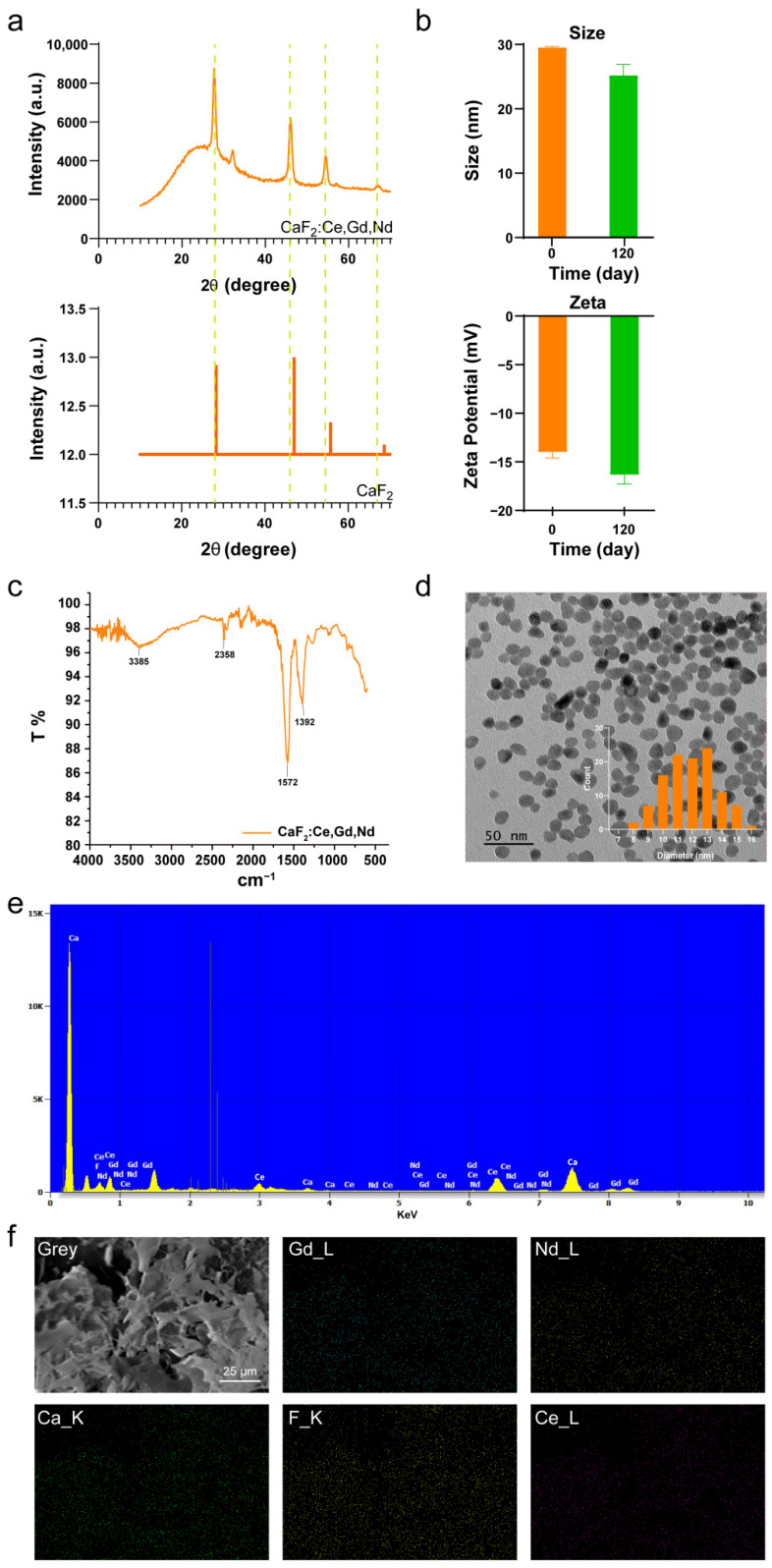
Morphology of CaF_2_: Ce, Gd, Nd NPs. (**a**) XRD images of CaF_2_: Ce, Gd, Nd NPs and CaF_2_; (**b**) DLS image of CaF_2_: Ce, Gd, Nd NPs at 0 d and 120 d; (**c**) FTIR image of CaF_2_: Ce, Gd, Nd NPs; (**d**) TEM images of CaF_2_: Ce, Gd, Nd NPs, where the inset shows the size distribution of CaF_2_: Ce, Gd, Nd NPs; (**e**) EDS spectrum of CaF_2_: Ce, Gd, Nd NPs; (**f**) mapping images of CaF_2_: Ce, Gd, Nd NPs.

First, to confirm that the main matrix of the synthesized NPs consisted of CaF_2_, we performed an XRD analysis (Figure 1a). Through XRD pattern analysis, it can be seen that the main diffraction peaks of CaF_2_: Ce, Gd, Nd powder correspond to the CaF_2_ crystal standard card, and the addition of Ce^3+^, Gd^3+^, Nd^3+^ did not change the CaF_2_ cubic phase structure but only resulted in a slight shift of diffraction peaks (Figure 1a). This is because the trivalent lanthanide ions replaced Ca^2+^, which resulted in Ca vacancies and exhibited lattice defects. Moreover, due to the differences in ionic radius and valence, the excess of positive charge needed more F^−^ to compensate, which changed the bond length and reduced the symmetry of the crystal [51]. In addition, a small peak appeared at about 30°, which has been demonstrated to correspond to the doping of rare-earth elements [52,53]. This also confirmed the successful doping of Ce^3+^, Gd^3+^, Nd^3+^ into the CaF_2_ crystal. DLS data showed that the NPs had a relatively stable particle size of 29.53 ± 0.16 nm in water. Zeta potential measurement showed that the NPs had a negative surface charge of −16.6 ± 0.75 mV. This is because sodium citrate was used as a complexing agent during NPs synthesis, and as a result, the NPs surface harbors carboxyl groups. The carboxyl group of citrate is negatively charged in water, and the electrostatic repulsion is the reason why CaF_2_: Ce, Gd, Nd NPs are stable in solution. To examine the stability and shelf-life of CaF_2_: Ce, Gd, Nd NPs, we repeated the DLS measurement after storing the NPs for 4 months at room temperature (Figure 1b). There was no significant change in the particle size and zeta potential of the NPs, suggesting that CaF_2_: Ce, Gd, Nd NPs show excellent shelf-life stability. CaF_2_: Ce, Gd, Nd NPs have two main absorption peaks at 1392 and 1572 cm^−1^, which correspond to the sodium citrate bands (Figure 1c). On the other hand, the absorption peaks, indicating that the carboxyl groups were conjugated to the metal ion on the surface of the NPs. Therefore, FTIR effectively proved the presence of citrate anions on the surface of the CaF_2_: Ce, Gd, Nd NPs [54]. In order to study the morphology of CaF_2_: Ce, Gd, Nd NPs more accurately, we performed a TEM analysis (Figure 1d). The TEM results showed that the NPs have a uniform and stable morphology, about 12 nm, which met our expectations. The discrepancy between the DLS and TEM results on particle size is due to the fact that the DLS measures the hydrodynamic diameter, while the TEM measures the hard boundary [55]. Additionally, to further investigate the elemental composition of the CaF_2_: Ce, Gd, Nd NPs, we performed EDS on powdered NPs. In Figure 1e, we can see that the strongest peak corresponds to Ca, the smaller peaks to F, Ce and Gd and the weakest peak to Nd. We did not detect any impurity peaks. This result is consistent with the proportion of doped elements in our synthesis process. In addition, mapping results revealed signals for all these elements, and the distribution is homogeneous (Figure 1f). In summary, these results prove that the rare-earth ions (Ce^3+^, Gd^3+^, Nd^3+^) have been successfully doped into CaF_2_.

**Figure 2:**
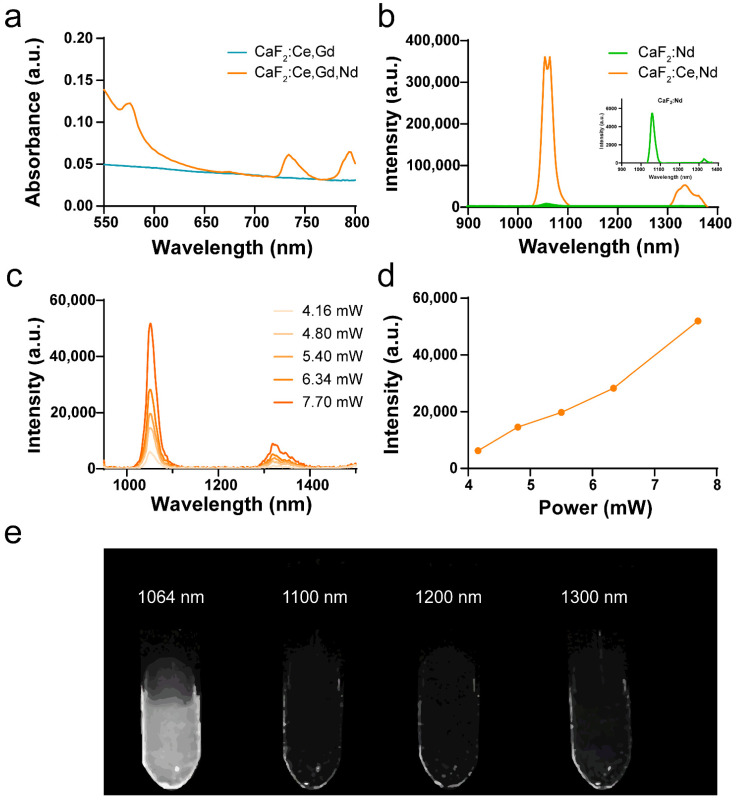
Optical properties of CaF_2_: Ce, Gd, Nd NPs. (**a**) The absorption spectra of CaF_2_: Ce, Gd, Nd NPs solution and CaF_2_: Ce, Gd NPs (10 mg/mL) measured with SpectraMax^®^ iD3 Multi-Mode Microplate Reader; (**b**) emission spectra of CaF_2_: Nd NPs and CaF_2_: Ce, Nd NPs (powder) measured with Edinburgh FLS920 fluorescence spectrometer with an 808 nm NIR diode laser (300 mW), where the inset shows a zoomed-in spectra of CaF_2_: Nd NPs; (**c**) emission spectra of CaF_2_: Ce, Gd, Nd NPs under 808 nm laser excitation; (**d**) the emission intensities of CaF_2_: Ce, Gd, Nd-NPs at different laser powers (excited by an 808 nm laser); (**e**) NIR-II images of CaF_2_: Ce, Gd, Nd NPs (2 mg/mL) at different wavelengths (excited by an 808 nm laser, 50 mW/cm^2^).

In order to evaluate the optical properties of the NPs, we first measured the absorption spectra of the CaF_2_: Ce, Gd, Nd NPs and CaF_2_: Ce, Gd NPs in aqueous solution at a concentration of 10 mg/mL (Figure 2a). Compared to CaF_2_: Ce, Gd NPs, the CaF_2_: Ce, Gd, Nd NPs showed absorption peaks at 576 nm,734 nm and 795 nm, corresponding to the ^4^G_5/2_+^2^G_7/2_, ^4^F_7/2_+^4^S_3/2_, and ^4^F_5/2_+^2^H_9/2_ absorption peaks of Nd^3+^ [45,56]. Then, to demonstrate that the doping of Ce increased the luminescence intensity of the NPs, we first tested the luminescence intensity of CaF_2_: Nd and CaF_2_: Ce, Nd under 808 nm laser excitation (300 mW) (Figure 2b). The experimental results demonstrated that the doping of Ce significantly increased the luminescence intensity of the NPs, and this result was consistent with the results of Wang et al. [47] Next, we measured the luminescence intensity of the NP sample under increasing laser powers at an excitation wavelength of 808 nm (Figure 2c). The NPs showed a strong signal in the near-infrared region, with two main emission peaks at 1056 nm and 1340 nm, corresponding to the ^4^F_3/2_ → ^4^I_11/2_ and ^4^F_3/2_ → ^4^I_13/2_ electronic transitions of Nd^3+^, respectively [57,58,59]. The strongest emission peak was observed at 1056 nm, when the laser power reached 7.7 mW, and an intensity of about 5×10^4^, which proves its unique NIR-II efficiency (Figure 2d). On the other hand, since Nd^3+^ is the main emission center of the NPs, under 808nm laser excitation, CaF_2_: Ce, Gd, Nd NPs can effectively reduce the biological tissue damage during the application process, especially the overheating effect caused by the laser. Moreover, the Nd^3+^ emission spectra were located in the second near-infrared window (1000–1400 nm), resulting in a deeper penetration depth and smaller autofluorescence effect of the NPs [52]. Based on the above generated data, an NIR-II system was employed to evaluate the NIR-II imaging properties of CaF_2_: Ce, Gd, Nd NPs (Figure 2e). Under 808 nm laser excitation, CaF_2_: Ce, Gd, Nd NPs showed near-infrared emission at 1064 nm. This result is consistent with the ^4^F_3/2_ → ^4^I_11/2_ energy transition of Nd^3+^, which proves that the main emission center is Nd^3+^. Thus, our data confirms that CaF_2_: Ce, Gd, Nd NPs are suitable NIR-II imaging probes.

**Figure 3:**
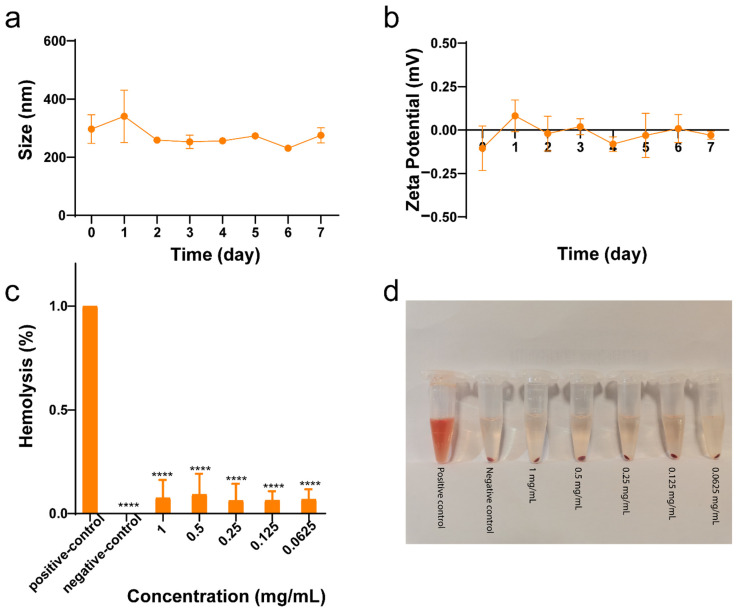
Stability of CaF_2_: Ce, Gd, Nd NPs. (**a**) Size of CaF_2_: Ce, Gd, Nd NPs in 50% FCS; (**b**) Zeta potential of CaF_2_: Ce, Gd, Nd NPs in 50% FCS; (**c**) hemolysis of CaF_2_: Ce, Gd, Nd NPs after incubation with red blood cells at multiple concentrations (0–1 mg/mL) for 4 h, with PBS and deionized water serving as negative and positive controls, respectively; (**d**) hemolysis photo after centrifugation. All data presented the mean values ± SD from three independent trials. By comparing experimental groups to control group, one-way ANOVA was used to determine statistical significance (**** *p* < 0.0001).

Good colloidal stability is one of the prerequisites for judging whether NPs can be used in biological applications. In order to determine whether CaF_2_: Ce, Gd, Nd NPs have good colloidal stability, the NPs were redispersed in NaCl containing 50% FCS, and then the particles were measured repeatedly (three times) using a Malvern ZetaSizer 2000 (Malvern, UK) at different time periods to determine the particle size. The results showed that there was no significant change in the size and zeta potential of the NPs in 50% FCS (Figure 3a,b). This demonstrates that the NPs exhibit a relatively stable state when 50% FCS was added to NaCl.

To evaluate the NPs’ biocompatibility with blood components, the effect of CaF_2_: Ce, Gd, Nd NPs on red blood cell (RBC) hemolysis was studied. To this end, murine peripheral blood cells were incubated at 37 °C for 4 h with different concentrations of CaF_2_: Ce, Gd, Nd NPs, and the hemolysis rate of the NPs was calculated by comparing the absorbance (λ = 540 nm) of the samples with the positive (1% Triton X-100) and negative (saline) controls [60] (Figure 3c,d). At the highest concentration (1 mg/mL), the hemolysis rate was calculated to be 0.08%, which is negligible. Thus, our results show that CaF_2_: Ce, Gd, Nd NPs have excellent blood compatibility and can be used for intravenous in vivo imaging.

**Figure 4:**
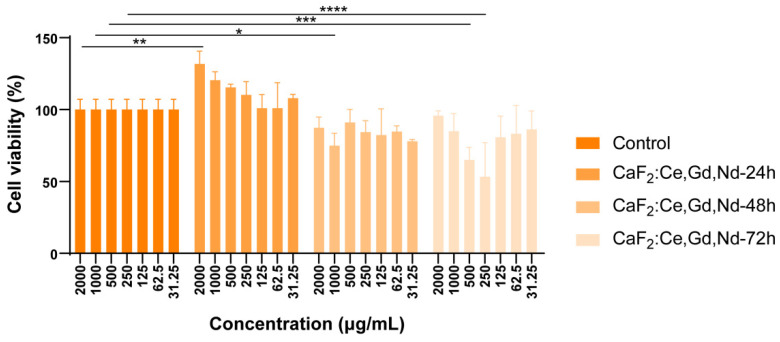
Cell viability. 4T1 cells were treated with CaF_2_: Ce, Gd, Nd NPs at varying concentrations (0–2000 µg/mL) for 24 h, 48 h and 72 h. Data represent the mean values ± SD from three independent experiments. Statistical significance was calculated using two-way ANOVA, by comparing experimental groups to control group (* *p*< 0.05, ** *p*< 0.01, *** *p* < 0.001 and **** *p* < 0.0001).

To determine whether the NPs induce cellular toxicity, the effect of CaF_2_: Ce, Gd, Nd NPs on the viability of 4T1 cells was measured by MTS assay (Figure 4). 4T1 is a highly tumorigenic and aggressive breast cancer cell line which grows and spreads metastatically, similar to human breast cancer, and is used as a typical cell model for cancer studies. 4T1 cells were cultured in the presence of different concentrations of CaF_2_: Ce, Gd, Nd NPs for 24 h, 48 h and 72 h. At 24 h, CaF_2_: Ce, Gd, Nd NPs below 1000 µg/mL had no significant effect on the viability of 4T1 cells (*p* > 0.05). Cell viability was significantly higher at a concentration of 2000 µg/mL (** *p* < 0.01). Values higher than 100% indicate that the NPs may have a positive effect on cell proliferation [61]. Secondly, CaF_2_: Ce, Gd, Nd NPs contain citric acid groups on the surface, which may participate in the intracellular tricarboxylic acid cycle reaction, resulting in increased cell activity. At 48 h, cell viability was significantly reduced by 15% when the concentration of CaF_2_: Ce, Gd, Nd NPs was 1000 µg/mL compared to the control (* *p*<0.05). At 72 h, the doses of 500 and 250 µg/mL CaF_2_: Ce, Gd, Nd NPs showed a significant inhibitory trend on 4T1 cells (*** *p* < 0.001, **** *p* < 0.0001). There was no difference between the cell survival rates when the concentration was < 125 µg/mL compared to the control group (*p* > 0.05). As cancer cell lines show biological differences compared to normal, healthy cells, we also tested the effect of CaF_2_: Ce, Gd, Nd NPs (0–125 µg/mL) on the viability of human peripheral blood mononuclear cells (PBMCs). As shown in Figure S1, there was no significant cytotoxic effect of the NPs on cell activity at 24 h and 48 h after NP incubation compared to the control group. Thus, CaF_2_: Ce, Gd, Nd NPs show great promise for biological applications, but further studies are needed to determine the optimal concentration and potential cytotoxic effects.

**Figure 5:**
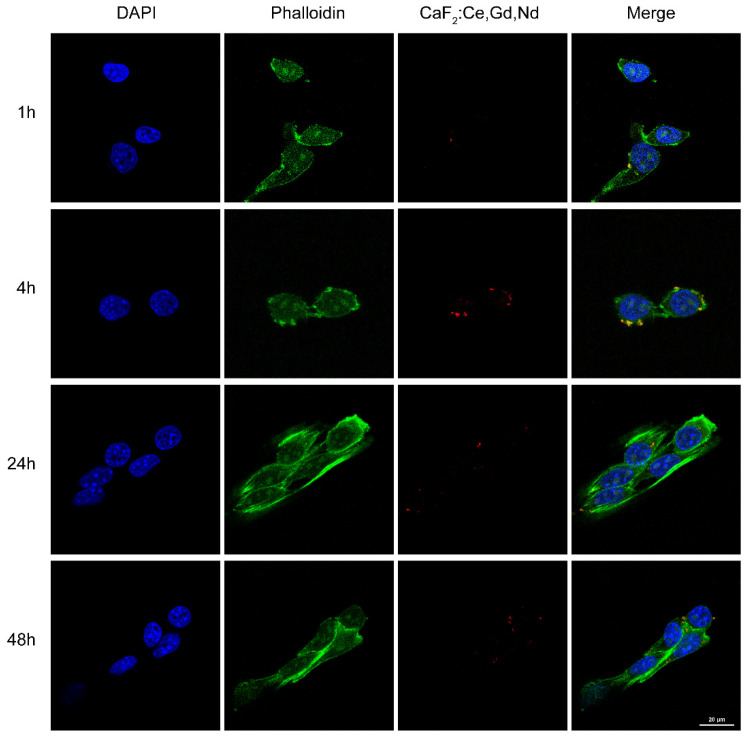
Uptake of CaF_2_: Ce, Gd, Nd NPs by 4T1 cells. Representative confocal microscopy images showing the uptake of CaF_2_: Ce, Gd, Nd NPs after 1 h, 4 h, 24 h and 48 h of incubation at 37 °C. Cell nuclei (blue), actin cytoskeleton (green), CaF_2_: Ce, Gd, Nd NPs (red). Scale bar = 20 µm.

We further investigated the uptake potential of CaF_2_: Ce, Gd, Nd NPs by murine breast cancer cells. As demonstrated in Figure 2a, optical characteristics of CaF_2_: Ce, Gd, Nd NPs overlap with the excitation and emission spectra of commonly employed dyes in flow cytometry and microscopy applications, such as Alexa Fluor 568. Thus, we reasoned that our NPs might be inherently monitorable by confocal microscopy. In order to determine the NP uptake rate and intracellular localization of NPs in 4T1 cells, we performed a confocal analysis (Figure 5). To this end, 4T1 cells were incubated with 250 µg/mL CaF_2_: Ce, Gd, Nd NPs (red) at 37 °C for 1 h, 4 h, 24 h and 48 h, followed by co-labeling with DAPI (blue) to stain the nucleus and phalloidin (green) to stain the actin cytoskeleton. Visual inspection confirmed that CaF_2_: Ce, Gd, Nd NPs could be effectively visualized as distinct dots by confocal microscopy. After 1 h co-incubation, few NPs were found attached to the cell membrane. With increasing incubation time, more red fluorescent dots were found in the cytoplasm, indicating that more NPs entered the cells (Figure 5). Based on the above results, we conclude that CaF_2_: Ce, Gd, Nd NPs were effectively taken up by 4T1 cancer cells.

**Figure 6:**
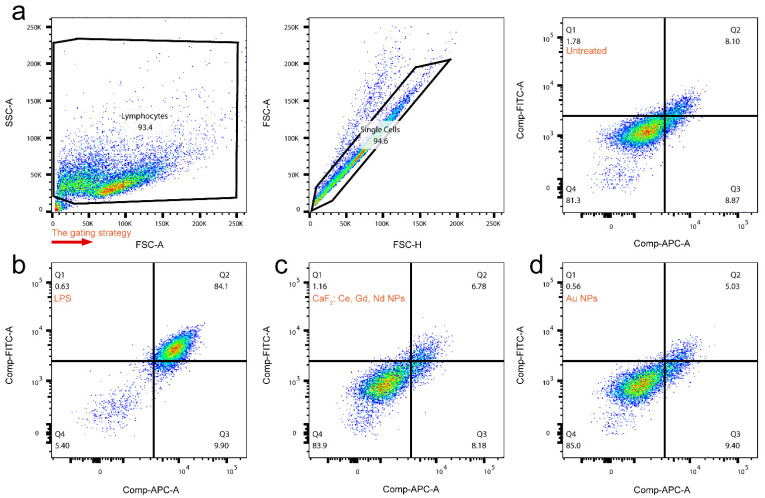
In vitro D1DC activation of CaF_2_: Ce, Gd, Nd NPs measured with FACS flow cytometry. D1DCs were incubated with samples for 24 h. (**a**) Gating strategy of D1DCs; (**b**) LPS (1 µg/mL) incubate with D1DCs as the positive control; (**c**) CaF_2_: Ce, Gd, Nd NPs (125 µg/mL) incubate with D1DCs; (**d**) Au NPs (125 µg/mL) incubate with D1DCs as the negative control.

Next, we determined whether CaF_2_: Ce, Gd, Nd NPs, without further modification, can activate the immune system, which could cause adverse side effects in vivo. Previous studies have shown that when immature dendritic cells (DCs) encounter various activation stimuli, they will mature and increase the expression of costimulatory markers on their surface [62,63,64]. Therefore, we cultured murine immature D1DCs in the presence of CaF_2_: Ce, Gd, Nd NPs for 24 h and assessed the expression of the DC costimulatory receptors CD86 and CD40 by flow cytometry. As positive control for DC maturation, we included D1 cells that were treated with LPS. After duplicate exclusion, the expression of CD86 and CD40 was analyzed on single D1 cells (Figure 6a). In the non-treated control group, 8% of the cells were double positive for CD86 and CD40 (Figure 6a), and the expression increased to 84% when the cells were treated with LPS (Figure 6b). When D1 cells were treated with a high concentration of CaF_2_: Ce, Gd, Nd NPs (125 µg/mL), 7% of the cells co-expressed CD86 and CD40 (Figure 6c), similar to the levels in the controls group, and in a negative controls group, where Au NPs were added that are known to be non-immunogenic (Figure 6d). In summary, these data indicate that CaF_2_: Ce, Gd, Nd NPs are inert and do not induce immune activation.

**Figure 7:**
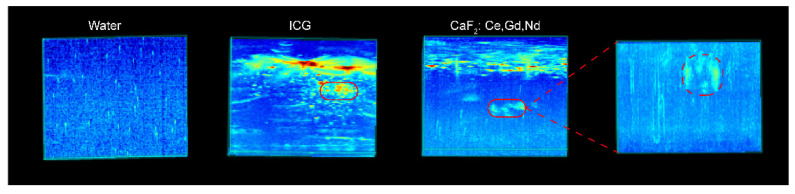
In vitro PA images of CaF_2_: Ce, Gd, Nd NPs (5 mg/mL) in the agarose gel.

Yang et al. have shown that rare-earth-doped particles can be employed as PAI agents [65]. Furthermore, since PAI is an imaging method that combines light excitation and ultrasound technology, the multiple absorption peaks of rare-earth NPs provide an opportunity as a PAI contrast agent. In order to determine whether CaF_2_: Ce, Gd, Nd NPs are suitable for ultrasound imaging, we placed the NPs into an agarose phantom, mimicking biological tissue, for imaging by PA (Figure 7). In this gel phantom, the NPs showed PA signals at 808 nm wavelength, which was stronger than in the control agarose without NPs, but slightly dimmer than in the ICG group, a dye that is well suited for PAI. When the NPs were irradiated by the laser, part of the light energy was absorbed and converted into heat energy, which caused thermoelastic expansion and generated the PA signal. It effectively proves that CaF_2_: Ce, Gd, Nd NPs have the ability to serve as NIR-II and PA probes.

**Figure 8:**
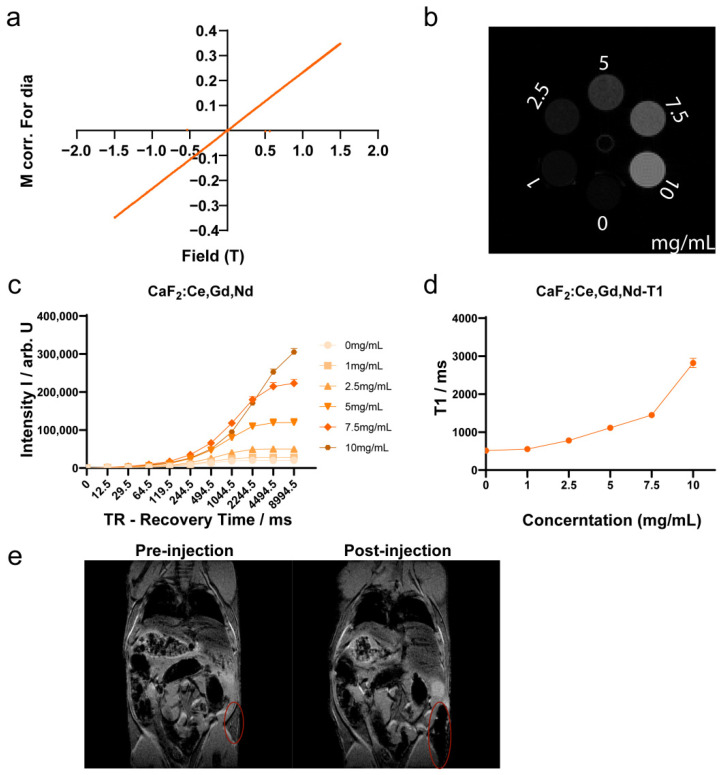
In vitro MRI performance of CaF_2_: Ce, Gd, Nd NPs. (**a**) Magnetic properties of CaF_2_: Ce, Gd, Nd NPs; (**b**) in vitro T1-weighted MRI of CaF_2_: Ce, Gd, Nd NPs at different concentrations in water containing 0.5 % agarose gel; (**c**) MRI signal intensity of CaF_2_: Ce, Gd, Nd NPs with increasing recovery time at different concentrations; (**d**) in vitro T1 relaxation rates of various CaF_2_: Ce, Gd, Nd NPs concentrations; (**e**) ex vivo MRI images of a mouse cadaver before and after subcutaneous injection of CaF_2_: Ce, Gd, Nd NPs (10 mg/mL).

Since Gd^3+^ chelating material is a common “positive” clinical MRI contrast agent, we inferred that CaF_2_: Ce, Gd, Nd NPs might be suitable as imaging probes for MRI. The magnetic properties of CaF_2_: Ce, Gd, Nd NPs were first verified using VSM. At room temperature (300 K) and an applied magnetic field of 1.5 T, we noticed that the NPs enhance their magnetic properties as the magnetic field increases, showing a typical paramagnetic behavior consistent with the magnetic characteristics of Gd ions (Figure 8a). The diamagnetic contribution was calculated to be 0.0032 Am^2^/kg [66,67]. In order to prove the hypothesis that CaF_2_: Ce, Gd, Nd NPs can be used as a MRI probe, we mixed CaF_2_: Ce, Gd, Nd NPs with agarose gel and performed an MRI measurement. Figure 8b shows that as the concentration of NPs increased, the MRI image became brighter. Analysis of the data showed that due to the increase in the concentration of CaF_2_: Ce, Gd, Nd NPs, the paramagnetic strength increased, which led to an increase in the longitudinal relaxation time (T1), resulting in a bright image on the T1 weighted imaging (Figure 8c,d). To better investigate the potential biological applications of NPs, we injected NPs subcutaneously into mouse cadavers and found a clear signal at the injection site (Figure 8e). These results indicate that CaF_2_: Ce, Gd, Nd NPs can be used as MRI contrast agents.

## 4. Conclusions

In summary, we doped Ce^3+^, Gd^3+^ and Nd^3+^ into CaF_2_ crystals through a simple hydrothermal process, resulting in the synthesis of CaF_2_: Ce, Gd, Nd NPs suitable for multimodal imaging. The synthesized NPs were highly pure, and showed low toxicity, good biocompatibility and no immunogenicity. CaF_2_: Ce, Gd, Nd NPs themselves exhibited dual modes because Ce^3+^ and Nd^3+^ dopants contribute to NIR-II and PAI, and the presence of Gd^3+^ shows a high-contrast T1-enhancing effect for MRI. Therefore, CaF_2_: Ce, Gd, Nd NPs may be an informative NIR-II/PA/MR multimodal probe for clinical diagnosis. This research also laid the foundation for the use of CaF_2_: Ce, Gd, Nd NPs for biological imaging of cells and deep tissues.

## Data Availability

Not applicable.

## Supplementary Materials

The following supporting information can be downloaded at: https://www.mdpi.com/article/10.3390/pharmaceutics14122796/s1, Figure S1: Cell viability. PBMCs were treated with CaF_2_: Ce, Gd, Nd NPs at varying concentrations (0–125 µg/mL) on PBMCs for 24 h and 48 h. Data represent the mean values ± SD from three independent experiments. Statistical significance was calculated using two-way ANOVA, by comparing experimental groups to control group.
