# Correction: Yu et al. Rare-Earth-Metal (Nd^3+^, Ce^3+^ and Gd^3+^)-Doped CaF_2_: Nanoparticles for Multimodal Imaging in Biomedical Applications. *Pharmaceutics* 2022, *14*, 2796

**DOI:** 10.3390/pharmaceutics15122755

**Published:** 2023-12-12

**Authors:** Zhenfeng Yu, Yuanyuan He, Timo Schomann, Kefan Wu, Yang Hao, Ernst Suidgeest, Hong Zhang, Christina Eich, Luis J. Cruz

**Affiliations:** 1 Translational Nanobiomaterials and Imaging Group, Department of Radiology, Leiden University Medical Center, 2333 ZA Leiden, The Netherlands; z.yu@lumc.nl (Z.Y.);; 2 Percuros B.V., Zernikedreef 8, 2333 CL Leiden, The Netherlands; 3 Van ‘t Hoff Institute for Molecular Sciences, University of Amsterdam, Science Park 904, 1098 XH Amsterdam, The Netherlands; 4 C.J. Gorter Center for High Field MRI, Department of Radiology, Leiden University Medical Center, 2333 ZA Leiden, The Netherlands

## 1. Figure Legend

In the original publication [1], there was a mistake in the legend for Figure 8. The legends of Figure 8b–d are inaccurate and need to be corrected. The correct legend appears below. The authors state that the scientific conclusions are unaffected. This correction was approved by the Academic Editor. The original publication has also been updated.

**Figure 8**. In vitro MRI performance of CaF_2_: Ce, Gd, Nd NPs. (**a**) Magnetic properties of CaF_2_: Ce, Gd, Nd NPs; (**b**) in vitro T_1_-weighted and T_2_-weighted MR images of CaF_2_: Ce, Gd, Nd NPs at different concentrations in water containing 1% agarose gel; (**c**) MRI signal intensity of CaF_2_: Ce, Gd, Nd NPs with increasing repetition time and echo time at different concentrations; (**d**) in vitro T_1_ relaxation rates and T_2_ relaxation rates of various Gd concentrations for CaF_2_: Ce, Gd, Nd NPs; (**e**) ex vivo MRI images of a mouse cadaver before and after subcutaneous injection of CaF_2_: Ce, Gd, Nd NPs (10 mg/mL).

## 2. Error in Figure

In the original publication, there was a mistake in Figure 8, as published. The original MRI data of Figure 8b–d were incomplete and not analyzed correctly. The corrected Figure 8 appears below. The authors state that the scientific conclusions are unaffected. This correction was approved by the Academic Editor. The original publication has also been updated.


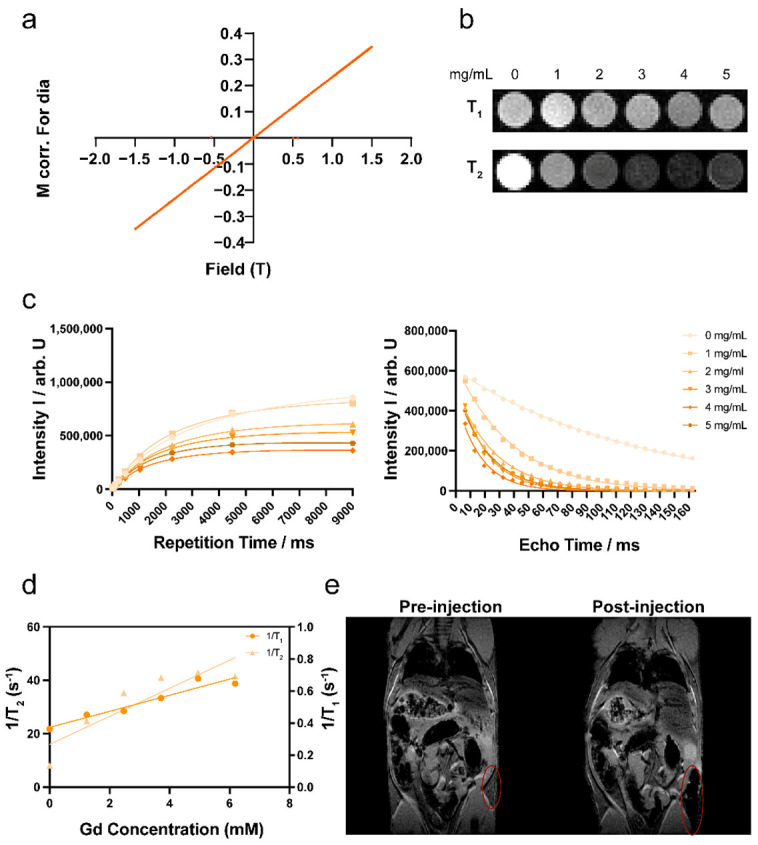


## 3. Text Correction

There was an error in the original publication. In the introduction, one word is missing in the full name of SPECT.

A correction has been made to 1. Introduction, Paragraph 1:

Imaging holds a crucial role in the diagnosis of a variety of diseases such as cancer. Early-stage disease diagnosis is important to maximize treatment effects, and to personalize treatments based on the patient’s individual variability and medical profile. Molecular imaging techniques provide comprehensive anatomical, physiological and functional information on disease detection and the monitoring of treatment responses. The most commonly used diagnostic imaging methods during the past few decades in the medical field include MRI, X-ray computed tomography (CT) [1], positron emission tomography (PET) [2,3], single-photon emission computed tomography (SPECT) [4], optical fluorescent light imaging (FLI) and photoacoustic imaging (PAI) [5]. Due to differences in their detection methods, spatiotemporal resolution, sensitivity and probe types, the diagnostic information obtained is divergent. Both PET and SPECT use γ rays to detect the in vivo distribution of radioactive tracers to obtain information on biological functions. They have the disadvantages of low spatial resolution, radiation risks and high costs [6–10]. Optical imaging uses visible light and near-infrared probes with different spectral characteristics for molecular and cellular detection but faces several limitations, such as photobleaching, low tissue penetration power, low spatial resolution and autofluorescence [11–15]. While CT, MRI and PAI can provide structural information, CT detection relies on contrast agents (such as iodine or barium) to obtain images through the different absorption of X-rays by biological tissues, which has the disadvantage of radiation risk and limited soft-tissue resolution [16,17]. MRI, a non-invasive imaging technology uses radio waves (magnetic field), but it has low sensitivity, high cost and scanning and image processing are time-consuming [18–20]. PAI uses high-frequency sound waves (>20 kHz) to generate acoustic energy to detect the difference in echo between chromophores or microbubbles and surrounding tissues in real time. However, due to the limited resolution and sensitivity, the data reproducibility is low, and it cannot provide accurate results [8,21–23]. In summary, to predict and treat diseases more comprehensively and accurately, it is imperative to develop a simple and efficient multifunctional nanomaterial that can integrate multiple imaging modes for detection.

There was an error in the original publication. There is a mistake in the description.

A correction has been made to 1. Introduction, Paragraph 4:

Since the radius of Nd^3+^ is not much different from that of Ca^2+^, trivalent Nd ions replace the crystal sites of divalent Ca ions when they are doped into the CaF_2_ lattice, requiring more F^−^ for charge compensation, but they do not cause obvious crystal changes. However, when the Nd^3+^ doping concentration reaches a certain limit, Nd^3+^ aggregates and some energy cross-relaxation occurs, which triggers the cluster effect and reduces the luminous efficiency of the nanomaterials [40,41]. In order to overcome this phenomenon, the introduction of optically inactive ions (such as Lu^3+^, Gd^3+^, Y^3+^, Yb^3+^) can effectively destroy the formation of Nd-Nd clusters, thereby improving the quantum efficiency of the material [42–45]. Notably, the special properties of certain optically inactive ions also offer the possibility of constructing multimodal probes; however, most current multimodal imaging probes are dual-mode probes, and NIR-II-based imaging systems combining more than two modes are still rarely reported, but our previous study demonstrates that triple-mode imaging probes hold great promise for obtaining complementary information. Therefore, we focus on the field of rare-earth triple-mode imaging probes for more exploration [46]. Wang et al. showed that the luminous efficiency of CaF_2_: Nd co-doped Ce^3+^ was 10.4 times higher than that of CaF_2_: Nd [47]. On the other hand, the seven unpaired electrons of Gd(III) can increase the proton relaxation rate, making Gd^3+^ a common contrast agent in MR bioimaging [48,49]. Therefore, we choose Ce^3+^, Gd^3+^ and Nd^3+^ co-doping CaF_2_ as the material in our strategy to construct an imaging mode combining NIR-II imaging with complementary MRI and PAI.

There was an error in the original publication. There is a need to correct some details.

A correction has been made to 2.9.3. MRI Studies:

MRI studies were carried out using a 7T Bruker BioSpec (Ettlingen, Germany), and ParaVision 360 (Version 2.0. pl.1) software was used to analyze attenuation images. CaF_2_: Ce, Gd, Nd NPs were dissolved in 1% agarose solution, and the concentrations were 0 mg/mL, 1 mg/mL, 2 mg/mL, 3 mg/mL, 4 mg/mL and 5 mg/mL. The microwave oven was used to heat the solutions and create the gel sample. T_1_ relaxation was assessed using a saturation recovery sequence and the parameters were as follows: repetition times (TR) array = 18, 35, 70, 125, 250, 500, 1050, 2250, 4500, 9000 ms; echo time (TE) = 6 ms; matrix size (MTX) = 64 × 64; field of view (FoV) = 30 × 30 mm^2^; slice thickness (SL) = 2 mm. T_2_ relaxation was measured using a multi spin echo sequence and the parameters were as follows: TR = 2200 ms; TE = 6.5 ms; echo spacing = 25 echoes; MTX = 64 × 64; FoV = 30 × 30 mm^2^ and SL = 2 mm. To investigate the MRI characteristics of NPs under biological conditions, we fixed C57BL/6J mouse cadavers after subcutaneously injecting 100 μL of CaF_2_: Ce, Gd, Nd NPs (10 mg/mL) and took MR images before and after the injection. The measurement conditions were gradient echo sequence with TR/TE = 10/2.8 ms, FoV = 40 × 40 mm^2^, matrix = 256 × 256. The MRI data were acquired using ParaVision 360 (Version 2.0. pl.1, Bruker, Germany) software. Standard mono-exponential functions were used to calculate relaxation times.

There was an error in the original publication. We discovered errors in the reporting and interpretation of the MRI properties of the nanoparticles.

A correction has been made to 3. Results and Discussion, Paragraph 10:

Since Gd^3+^ chelating material is a common “positive” clinical MRI contrast agent, we inferred that CaF_2_: Ce, Gd, Nd NPs might be suitable as imaging probes for MRI. The magnetic properties of CaF_2_: Ce, Gd, Nd NPs were first verified using VSM. At room temperature (300 K) and an applied magnetic field of 1.5 T, we noticed that the NPs enhance their magnetic properties as the magnetic field increases, showing a typical paramagnetic behavior consistent with the magnetic characteristics of Gd ions (Figure 8a). The diamagnetic contribution was calculated to be 0.0032 Am^2^/kg [66,67]. In order to prove the hypothesis that CaF_2_: Ce, Gd, Nd NPs can be used as an MRI probe, we mixed CaF_2_: Ce, Gd, Nd NPs with agarose gel and performed an MRI measurement. Figure 8b shows that as the concentration of NPs increased, the T_2_-weighted MR image became darker; in this concentration range the visually observable contrast in T_1_-weighted MR images was much smaller. We investigated the MRI properties of the NPs by testing the longitudinal magnetization recovery and the transverse magnetization decay (Figure 8c). We plotted the R_1_ (1/T_1_) and R_2_ (1/T_2_) relaxation rates, the r_1_ and r_2_ relaxivity values were calculated (r_1_ = 0.05 mM Gd^−1^·s^−1^, r_2_ = 5.3 mM Gd^−1^·s^−1^). Notably, the NPs may tend to aggregate in the gel, which may account for the non-linear behaviour; therefore the relaxivities should be interpreted with some caution (Figure 8d). Nevertheless, r_2_/r_1_ was much greater than 10, therefore, the CaF_2_: Ce, Gd, Nd NPs could be considered as a T_2_ contrast agent [68]. To better investigate the potential biological applications of NPs, we injected NPs subcutaneously into mouse cadavers and found a clear signal at the injection site (Figure 8e). These results indicate that CaF_2_: Ce, Gd, Nd NPs can be used as MRI contrast agents.

There was an error in the original publication. The conclusion of the MRI performance of nanoparticles is not accurate.

A correction has been made to 4. Conclusions:

In summary, we doped Ce^3+^, Gd^3+^ and Nd^3+^ into CaF_2_ crystals through a simple hydrothermal process, resulting in the synthesis of CaF_2_: Ce, Gd, Nd NPs suitable for multimodal imaging. The synthesized NPs were highly pure, and showed low toxicity, good biocompatibility and no immunogenicity. CaF_2_: Ce, Gd, Nd NPs themselves exhibited dual modes because Ce^3+^ and Nd^3+^ dopants contribute to NIR-II and PAI, and the presence of Gd^3+^ shows a high-contrast T_2_ effect for MRI. As such it acts similarly to a super-paramagnetic agent, similar to results obtained for Gd_2_O_3_-mesoporous silica/gold nanoshells [68]. Therefore, CaF_2_: Ce, Gd, Nd NPs may be an informative NIR-II/PA/MR multimodal probe for clinical diagnosis. This research also laid the foundation for the use of CaF_2_: Ce, Gd, Nd NPs for biological imaging of cells and deep tissues.

The authors state that the scientific conclusions are unaffected. This correction was approved by the Academic Editor. The original publication has also been updated.

## 4. References

We added new reference 68. The authors state that the scientific conclusions are unaffected. This correction was approved by the Academic Editor. The original publication has also been updated.

68. Kadria-Vili, Y.; Neumann, O.; Zhao, Y.; Nordlander, P.; Martinez, G.V.; Bankson, J.A.; Halas, N.J. Gd_2_O_3_-mesoporous silica/gold nanoshells: A potential dual T_1_/T_2_ contrast agent for MRI-guided localized near-IR photothermal therapy. *Proc. Natl. Acad. Sci. USA* **2022**, *119*, e2123527119.
