# Supplementary material for: Rare-Earth-Metal (Nd3+, Ce3+ and Gd3+)-Doped CaF2: Nanoparticles for Multimodal Imaging in Biomedical Applications
Source: Pharmaceutics. 2022 Dec 14;14(12):2796. doi: 10.3390/pharmaceutics14122796 (PMC9784532; doi:10.3390/pharmaceutics14122796)
Supplement: Supplementary file 1 [file pharmaceutics-14-02796-s001.zip › pharmaceutics-1990998-figure s1.pdf]

pharmaceutics

Supporting Information:

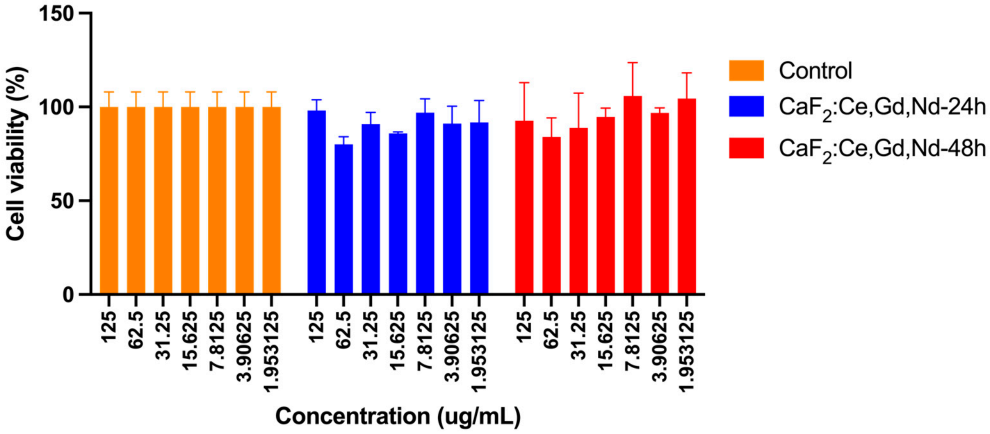

Figure S1. Cell viability. PBMCs were treated with CaF_2: Ce, Gd, Nd NPs at varying concentrations (0-125 μg/mL) on PBMCs for 24 h and 48 h. Data represent the mean values ± SD from three independent experiments. Statistical significance was calculated using two-way ANOVA, by comparing experimental groups to control group.
